# Association of High Myopia with Crystallin Beta A4 (*CRYBA4*) Gene Polymorphisms in the Linkage-Identified *MYP6* Locus

**DOI:** 10.1371/journal.pone.0040238

**Published:** 2012-06-29

**Authors:** Daniel W. H. Ho, Maurice K. H. Yap, Po Wah Ng, Wai Yan Fung, Shea Ping Yip

**Affiliations:** 1 Centre for Myopia Research, School of Optometry, The Hong Kong Polytechnic University, Hong Kong SAR, China; 2 Department of Health Technology and Informatics, The Hong Kong Polytechnic University, Hong Kong SAR, China; Central China Normal University, China

## Abstract

**Background:**

Myopia is the most common ocular disorder worldwide and imposes tremendous burden on the society. It is a complex disease. The *MYP6* locus at 22 q12 is of particular interest because many studies have detected linkage signals at this interval. The *MYP6* locus is likely to contain susceptibility gene(s) for myopia, but none has yet been identified.

**Methodology/Principal Findings:**

Two independent subject groups of southern Chinese in Hong Kong participated in the study an initial study using a discovery sample set of 342 cases and 342 controls, and a follow-up study using a replication sample set of 316 cases and 313 controls. Cases with high myopia were defined by spherical equivalent ≤ -8 dioptres and emmetropic controls by spherical equivalent within ±1.00 dioptre for both eyes. Manual candidate gene selection from the *MYP6* locus was supported by objective *in silico* prioritization. DNA samples of discovery sample set were genotyped for 178 tagging single nucleotide polymorphisms (SNPs) from 26 genes. For replication, 25 SNPs (tagging or located at predicted transcription factor or microRNA binding sites) from 4 genes were subsequently examined using the replication sample set. Fisher *P* value was calculated for all SNPs and overall association results were summarized by meta-analysis. Based on initial and replication studies, rs2009066 located in the crystallin beta A4 (*CRYBA4*) gene was identified to be the most significantly associated with high myopia (initial study: *P* = 0.02; replication study: *P* = 1.88e-4; meta-analysis: *P* = 1.54e-5) among all the SNPs tested. The association result survived correction for multiple comparisons. Under the allelic genetic model for the combined sample set, the odds ratio of the minor allele G was 1.41 (95% confidence intervals, 1.21-1.64).

**Conclusions/Significance:**

A novel susceptibility gene (*CRYBA4*) was discovered for high myopia. Our study also signified the potential importance of appropriate gene prioritization in candidate selection.

## Introduction

Myopia is present if distant objects are focused in front of, rather than on, the retina. It is the most common eye problem in the world. Its prevalence varies among populations with substantially higher prevalence in Asian populations than in Caucasian populations [1]–[3]. In particular, the prevalence of myopia in Hong Kong has increased considerably in the past few decades with the majority of Hong Kong Chinese suffering from this disorder, especially the younger generation. High myopia, often defined as a refractive error of -6.00 dioptres (D) or worse, severely elevates the risk of various degenerative eye diseases and is the leading cause of vision loss or even irreversible blindness [4]. It will thus potentially impose economic burden on Hong Kong society and working population in the long term. Despite easy and accurate diagnosis of myopia, prevention of myopia and its associated complications has not yet been realized because the underlying molecular pathological mechanism is still unclear.

Myopia is a common complex disease. The heritability of refractive error has been estimated to be ∼80-90% in several twin studies [5]–[8]. Such high heritability highlights the importance of genetic influence in myopia and justifies studying the genetics of myopia. Environmental factors are also important in myopia development and various environmental factors have also been postulated with excessive near work being regarded as the most prominent one [9].

To date, almost 20 myopia loci have been identified by linkage analyses (OMIM; http://omim.org/) [10], [11]. Of these, *MYP6* is of particular interest. *MYP6* was first mapped to 22 q12.1 (D22S689) by genome-wide linkage analysis involving 44 large American families of Ashkenazi Jewish descent [12]. In a follow-up study with 19 additional Jewish families, peak linkage evidence was found at 22 q12.3 (D22S685) [13]. Combined analysis of both studies confirmed the linkage of *MYP6* to 22 q12.1 (D22S689). Moreover, another genome-wide linkage study of the subjects from the Beaver Dam Eye Study also identified linkage evidence at 22 q [14]. Peak evidence was detected at 22 q11.23 (D22S345), but the results also supported a region of linkage from 22 q11.23 (D22S345) to 22 q12.3 (D22S685). Linkage signals at 22 q12.3 (rs2056965 and rs972153) and 22 q13.2 (rs139027) were also detected in yet another genome-wide linkage study [15]. Recently, linkage signal from the Beaver Dam Eye Study was further refined to 22 q11 [16]. Despite the strong evidence from these studies, no specific gene has been identified in this locus for myopia susceptibility.

The current study adopted a positional candidate gene approach to identifying myopia susceptibility gene through testing of candidate genes at this linkage-based *MYP6* locus. Biologically relevant candidate genes were carefully selected from this locus, and the selection was supported by independent computational gene prioritization via an *in silico* bioinformatics tool (Endeavour) [17]. Case-control association studies of single nucleotide polymorphisms (SNPs) were then carried out in two stages: an initial study of tag SNPs from HapMap [18], [19] for a discovery sample set (n = 684), and a replication study of suggestive and additional SNPs for an independent replication sample set (n = 629). Based on the analysis results, we found crystallin beta A4 (*CRYBA4*) to be a novel gene for myopia susceptibility.

## Results

### Analysis of Phenotype Data

Two groups of subjects were recruited for the case-control association studies. The first group of subjects (discovery set) consisted of 342 cases and 342 controls. The second group (replication set) had 316 cases and 313 controls. Cases with high myopia were defined by spherical equivalent (SE) ≤-8.00 D for both eyes, and controls by SE within ±1.00 D for both eyes. Table 1 summarizes the phenotypes for subjects in both groups. The ocular data reported herein were for right eyes as both eyes had very similar phenotype measurements.

**Table 1:** Characteristics of study subjects.

	Discovery sample set				Replication sample set			
Measurements - mean (SD)*	Cases (n = 342)		Controls (n = 342)		Cases (n = 316)		Controls (n = 313)	
Age, years	32.98	(8.89)	31.70	(9.43)	28.53	(7.52)	25.82	(7.14)
Proportion of females, %	70.47		58.48		68.35		56.23	
Spherical equivalent, D	−10.15	(2.41)	0.07	(0.54)	−10.63	(2.63)	0.02	(0.42)
Axial length, mm	27.59	(2.90)	23.75	(0.82)	27.80	(1.16)	23.83	(0.83)
Corneal power, D	44.92	(1.48)	44.16	(1.52)	44.84	(1.44)	43.89	(1.59)
Anterior chamber depth, mm	3.40	(0.41)	3.28	(0.41)	3.66	(0.35)	3.57	(0.34)
Lens thickness, mm	4.27	(0.55)	4.27	(0.62)	4.04	(0.52)	3.99	(0.53)

* All measurements are the mean values for the right eyes with the standard deviation (SD) shown in brackets. The only exception is the proportion of females in the subject groups, which is indicated as a percentage (%).

### Candidate Gene Selection and Validation by Endeavour

From the *MYP6* locus, 664 genes were retrieved. Based on literature search and biological relevance, *manual* prioritization categorized these genes into five categories (Table 2). In particular, there were 26 “highly relevant” genes. Based on Endeavour, objective *in silico* prioritization produced a global ranking for each gene. Mean rankings were computed for manually prioritized categories. The mean ranking for the “highly relevant” genes was found to be the highest among the five categories (Table 2). This result supported the subjective judgement by manual prioritization, and hence the genes in the “highly relevant” category were justified to be examined by subsequent case-control association studies.

**Table 2:** Prioritization of 664 genes in the *MYP6* locus.

Manual prioritization		Computational prioritization by Endeavour	
Category	No. of genes	Mean ranking for category	SD
Highly relevant genes	26	90.4	97.2
Possibly related genes	21	185.7	178.2
Unlikely genes	340	247.6	160.7
Homologous genes	137	327.0	215.3
Pseudo, putative or hypothetical genes	140	493.1	132.5

SD stands for standard deviation.

### Initial Study of 26 Candidate Genes using the Discovery Sample Set

In total, 178 SNPs were selected from 26 “highly relevant” candidate genes and genotyped for association testing with high myopia. Of these, 12 were discarded due to low genotype call rate (<80%) or lack of HWE in controls (*P*<0.001) (Table S1). There remained 166 markers from 25 candidate genes for subsequent analysis. Based on single-marker analysis, 25 SNPs were found to show suggestive significance (*P*<0.05) under at least one of the genetic models. In particular, only two SNPs remained significant after correction for multiple comparisons by permutations of case-control status of the subjects (Table 3): rs2800960 of *DGCR2* (best *P* = 4.86e-19 under dominant model) and rs4616572 of *PVALB* (best *P* = 4.86e–106) (empirical *P* = 1.00e-06 for 10,000,000 permutations; not shown in Table 3). However, the extremely skewed genotype distribution in controls or cases suggested that there might be genotyping errors involved.

**Table 3:** Single-marker analysis of SNPs from 4 genes on chromosome 22 by PLINK.

						Discovery sample set (342 cases and 342 controls)							Replication sample set (316 cases and 313 controls)							
			Allele*			Genotype counts (22/12/11)					Fisher’s exact tests		Genotype counts (22/12/11)					Fisher’s exact tests		
Gene†	SNP†	Physicalposition(bp)‡	2	1	MAF(HapMapCHB)	Cases	MAF	Controls	MAF	*P* (HWEincontrols)	Best*P* value	Model	Cases	MAF	Controls	MAF	*P* (HWEincontrols)	Best *P*value	Model	*P*_emp_ §
***DGCR2***	rs2238754^#^	19058146	T	C	0.202	20/126/191	0.246	16/122/202	0.226	0.758	0.406	A	18/124/163	0.263	15/108/181	0.227	1	0.141	D	0.974
	rs2800960 ¶+	19077049	T	C	0.226	0/55/265	0.086	0/0/337	0	1	**4.86e-19**	D	8/67/223	0.139	16/71/208	0.175	0.007	0.11	A	0.921
	rs12233351^T/M^	19117892	C	T	0.220	–	–	–	–	–	–	–	8/71/236	0.138	12/76/215	0.165	0.141	0.204	A	0.996
***GP1BB***	rs12165395^T^	19702781	C	T	0.024	–	–	–	–	–	–	–	0/14/302	0.022	1/5/307	0.011	0.033	0.11	D	0.939
	rs3810596 ¶^T/M+^	19710461	G	C	0.022	0/8/332	0.012	0/1/341	0.001	1	**0.02**	D	0/12/304	0.019	1/4/306	0.010	0.024	0.138	D	0.973
***CRYBA4***	rs5761635^T^	27014467	T	C	0.381	48/172/121	0.393	38/164/137	0.354	0.342	0.145	A	50/160/102	0.417	39/132/137	0.341	0.447	**0.003**	D	0.083
	rs2283843^T^	27017300	T	G	0.387	–	–	–	–	–	–	–	53/160/97	0.429	42/131/133	0.351	0.315	**0.002**	D	0.06
	rs5997109^T^	27017435	G	C	0.452	83/166/90	0.490	78/175/86	0.488	0.588	0.718	R	79/155/68	0.518	77/133/95	0.470	0.029	**0.017**	D	0.392
	rs2071860	27019128	T	C	0.387	–	–	–	–	–	–	–	48/160/104	0.410	41/126/138	0.341	0.162	**0.003**	D	0.083
	rs2071861 ¶	27021189	A	G	0.411	93/169/75	0.527	70/174/96	0.462	0.663	**0.019**	A	93/160/54	0.564	85/130/93	0.487	0.006	**3.10e-04**	D	**0.01**
	rs2071862	27021401	A	G	0.429	–	–	–	–	**–**	**–**	**–**	54/142/154	0.357	96/112/141	0.436	**2.03e-10**	–		–
	rs4276	27021425	A	G	0.363	–	–	–	–	**–**	**–**	**–**	46/165/104	0.408	37/136/138	0.338	0.704	**0.004**	D	0.117
	rs2239832	27021936	A	G	0.411	–	–	–	–	**–**	**–**	**–**	91/167/54	0.559	84/131/96	0.481	0.006	**7.99e-05**	D	**0.003**
	rs5752359	27025879	C	T	0.522	–	–	–	–	**–**	**–**	**–**	34/149/130	0.347	63/125/125	0.401	0.003	**0.002**	R	0.055
	rs2009066	27029545	G	A	0.411	–	–	–	–	**–**	**–**	**–**	92/159/58	0.555	70/126/102	0.446	0.014	**2.04e-05**	D	**7.79e-04**
	rs1018833^T^	27049011	T	C	0.327	–	–	–	–	**–**	**–**	**–**	36/140/133	0.343	28/123/152	0.295	0.679	0.076	A	0.865
	rs739310^T^	27051299	G	A	0.351	–	–	–	–	**–**	**–**	**–**	31/138/145	0.318	48/128/134	0.361	0.065	**0.041**	R	0.665
***PVALB***	rs9610583^#^	37193986	C	A	0.280	33/147/161	0.312	26/139/177	0.279	1	0.192	A	28/131/150	0.303	20/131/157	0.278	0.323	0.293	R	1
	rs4616572 ¶+	37194716	A	C	0.100	5/336/0	0.507	3/91/243	0.144	0.118	**4.86e-106**	D	3/58/245	0.105	3/46/241	0.090	0.488	0.344	D	1
	rs4821529	37196448	T	C	0.100	–	–	–	–	**–**	**–**	**–**	3/60/250	0.105	4/48/258	0.090	0.293	0.303	D	1
	rs2022068 ¶	37199806	G	A	0.304	37/146/156	0.324	14/158/168	0.274	0.002	**0.001**	R	30/125/157	0.296	18/144/145	0.293	0.027	0.098	R	0.917
	rs12171125^#^	37201450	G	A	0.119	8/85/247	0.149	3/78/259	0.124	0.448	0.206	A	12/64/234	0.142	4/58/243	0.108	0.765	0.073	R	0.858
	rs2284021^#^	37201817	T	C	0.185	9/104/226	0.180	7/87/245	0.149	1	0.133	D	3/87/224	0.148	5/99/207	0.175	0.113	0.219	A	0.997
	rs2284024	37204229	G	T	0.100	–	–	–	–	**–**	**–**	**–**	1/64/248	0.105	5/54/253	0.103	0.346	0.123	R	0.96
	rs4820254 ¶	37206341	G	T	0.179	14/120/208	0.216	5/90/247	0.146	0.392	**0.001**	A	18/75/222	0.176	7/74/211	0.151	0.82	**0.043**	R	0.676

* The major allele in the control group is designated as allele 1, and the minor allele as allele 2 unless follows allele designation in initial study.

† Four genes that showed suggestive significance in set-based test in the discovery sample set (see Table 4) were followed up with the replication sample set. Six SNPs within these four genes with nominal *P*<0.05 (marked by ¶) were first chosen for follow-up. Nineteen more SNPs were also selected for follow-up for the following reasons: (i) in LD with these six SNPs (not marked by any symbol); (ii) with potential functional relevance – T for SNP located at predicted transcription factor binding site, and M for SNP located at predicted microRNA binding site); or (iii) forming significantly associated haplotype windows with one of the six chosen SNPs (marked by #) – rs2238754 (#) and rs2800960 (¶) with normal *P* = 5.19e-13; rs9610583 (#) and rs4616572 (¶) with nominal *P* = 9.32e-46; and rs1217125 (#), rs2284021 (#) and rs4820254 (¶) with nominal *P* = 1.06e-07. As such, 13 SNPs that had not been genotyped in the discovery sample set are shown as missing data (−).

‡ SNPs are listed according to their sequential physical positions on chromosome 22 (NCBI build 37.1).

§ Empirical *P* values (*P*_emp_) are estimated based on 10,000,000 permutations. In each round of permutation (swapping of the case-control status), the best *original* result of every SNP is compared against the best result of the three tests (allelic, dominant and recessive) of that SNP, and also against the best results from all SNPs.

+ SNPs tested to have significant difference (*P*<0.05) in minor allele frequency (MAF) for control subjects between the discovery and the replication sample sets.

**Table 4:** Set-based association tests for genes in the *MYP6* locus.

Gene set	NSNP*	NSIG*	ISIG*	*P*_emp_ †
**Initial study**				
*PEX26*	3	0	0	1.000
*DGCR6*	7	0	0	1.000
*DGCR5*	7	1	1	0.097
*DGCR2*	12	1	1	**1.00e-05**
*DGCR14*	8	0	0	1.000
*GP1BB*	1	1	1	**0.012**
*ARVCF*	15	10	9	0.099
*MMP11*	3	0	0	1.000
*ADORA2A*	5	0	0	1.000
*CRYBB3*	7	0	0	1.000
*CRYBB2*	9	0	0	1.000
*HPS4*	6	1	1	0.159
*CRYBB1*	6	0	0	1.000
*CRYBA4*	3	1	1	**0.032**
*XBP1*	1	0	0	1.000
*NF2*	10	1	1	0.192
*OSM*	4	0	0	1.000
*SMTN*	3	0	0	1.000
*TIMP3*	13	0	0	1.000
*HMOX1*	3	1	1	0.122
*PVALB*	13	3	3	**1.00e-05**
*IL2RB*	19	0	0	1.000
*SOX10*	1	0	0	1.000
*PDGFB*	7	0	0	1.000
*ADRBK2*	1	0	0	1.000
**Replication study**				
*DGCR2*	3	0	0	1.000
*GP1BB*	2	0	0	1.000
*CRYBA4*	11	8	4	**0.009**
*PVALB*	8	0	0	1.000

* NSNP, NSIG and ISIG denote the number of SNPs in set, the number of SNPs with nominal *P*<0.05, and the number of *independent* SNPs (r^2^>0.8) with nominal *P*<0.05, respectively.

† Empirical *P* values (*P*_emp_) are estimated based on 100,000 permutations. Note that permutation is performed for correcting multiple comparisons of *independent* SNPs located within a given gene and tested by chi-squared test.

Set-based tests were also performed on marker sets defined by individual candidate genes. In set-based analysis, each gene was represented by a set of SNPs located within the gene interval and LD among SNPs within a gene was also taken in account. Hence, 25 marker sets were constructed from 166 SNPs of the 25 candidate genes. Marker sets from four genes (*DGCR2*, *GP1BB*, *CRYBA4* and *PVALB*) were found to show suggestive significance (*P*<0.05, Table 4). Therefore, these four genes were of particular interest and their corresponding markers with nominal *P*<0.05 (6 SNPs in total) under at least one of the genetic models were chosen for follow-up with a replication sample set. Since the initial study was to identify potentially associated markers for follow-up, 19 other SNPs from these four genes were also genotyped in the replication phase for the following reasons: in LD with these 6 suggestive markers, with potential functional relevance, or forming significantly associated haplotype windows with one of these 6 suggestive SNPs selected in the discovery sample set (see footnotes to Table 3 for details).

### Replication Study of 4 Genes using the Replication Sample Set

In the follow-up study, 25 SNPs from the 4 suggestive candidate genes were genotyped for the replication sample set (Table 3). One SNP (rs2071862) of *CRYBA4* was removed from association analysis because it was not in HWE in the controls. With a threshold of *P*<0.05, there were 10 SNPs from *CRYBA4* and 1 SNP from *PVALB* showing nominal significance under at least one genetic model. Of these, three SNPs (rs2071861, rs2239832 and rs2009066) from *CRYBA4* remained significant after correction for multiple testing (*P*<0.05 using 10,000,000 permutations). Note that rs2071861 was only nominally significant (*P* = 0.019, Table 3) in the discovery phase. All these three SNPs showed the highest significance under the dominant genetic model in the replication sample set with rs2009066 being the most significantly associated marker with high myopia (dominant model: nominal *P* = 2.04e-5, empirical *P* = 7.79e-4). Set-based tests were also undertaken, with only *CRYBA4* showing statistical significance (empirical *P* = 9.38e-3, Table 4).

### LD Pattern and Haplotype Analysis of *CRYBA4*

**Figure 1:**
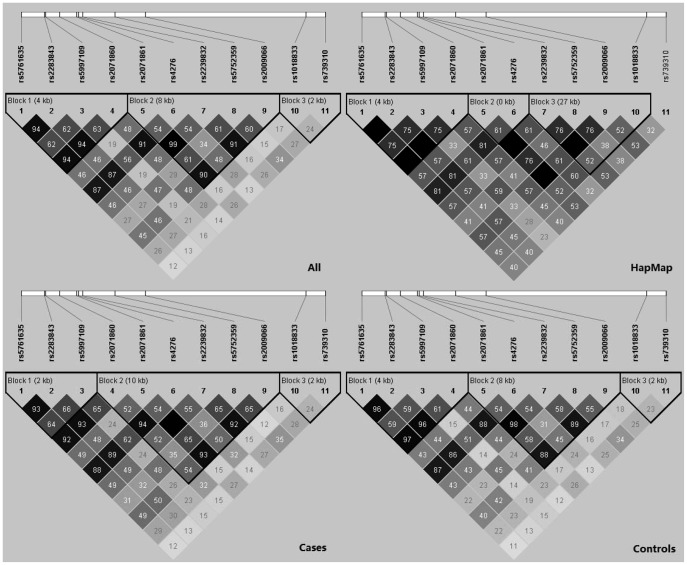
Linkage disequilibrium (LD) pattern across 11 single nucleotide polymorphisms of the *CRYBA4* gene. The LD patterns are for four different groups of subjects: all subjects (cases and controls combined) of the replication sample set, the Han Chinese of the HapMap database, cases of the replication sample set and controls of the replication sample set. LD measure is displayed as r^2^ value. LD blocks are defined by confidence bounds [20].

LD measures were calculated for 11 *CRYBA4* SNPs genotyped for the replication sample set (Figure 1). There were 3 haplotype blocks defined by confidence bounds [20] with sizes 4 kb, 8 kb and 2 kb respectively. All the 3 most significantly associated markers (rs2071861, rs2239832 and rs2009066) were located in the second haplotype block with strong LD among them. Under this haplotype block definition, haplotype association analysis identified the haplotype AAATG of block 2 to be significantly associated with high myopia (nominal *P* = 0.002 and empirical *P* = 0.017, Table 5). In addition, haplotypes were also examined by an exhaustive variable-sized sliding window strategy. There were a total of 55 windows with 2 to 11 SNPs per window, and 10 of these windows showed significant association with high myopia (empirical *P*<0.05, details not shown). The *best* sliding window was a 2-SNP window built by rs5752359 and rs2009066 (nominal *P* = 9.17e-4 and empirical *P* = 0.006, both omnibus test; Table 5) and the most significantly associated haplotype was TG (nominal *P* = 3.80e-4 and empirical *P* = 0.012, Table 5).

**Table 5:** Haplotype association analysis of *CRYBA4* SNPs for the replication sample set.

		Haplotype frequencies in				
Haplotype Block*	Haplotype*	Cases	Controls	OR	*P*_nom_ †	*P*_emp_ †
**Haploview: Block-based**						
Block 1	CGCC (1111)	0.474	0.530	0.80	**0.048**	0.437
(S1-S4-S3-S4)	TTGT (2222)	0.408	0.341	1.33	**0.014**	0.109
	CGGC (1121)	0.095	0.119	0.78	0.172	0.892
Block 2	GGGCA (11121)	0.340	0.392	0.80	0.056	0.540
(S5-S6-S7-S8-S9)	AAATG (22212)	0.400	0.316	1.44	**0.002**	**0.017**
	AGATG (21212)	0.149	0.141	1.07	0.697	1.000
	GGGTA (11111)	0.093	0.124	0.72	0.080	0.623
	AAATA (22211)	0.008	0.018	0.44	0.116	0.723
Block 3	CA (11)	0.338	0.342	0.98	0.871	1.000
(S10-S11)	CG (12)	0.318	0.361	0.83	0.105	0.693
	TA (21)	0.344	0.296	1.25	0.070	0.591
**Plink: best sliding-window**						
S8-S9	Omnibus	–	–	–	**9.17e-04**	**0.006**
	CA (21)	0.3480	0.4094	0.77	**0.032**	0.631
	TA (11)	0.0992	0.1443	0.65	**0.018**	0.421
	TG (12)	0.5528	0.4463	1.54	**3.80e-04**	**0.012**

* Haplotypes are indicated in both the ACGT and the 1–2 (major-minor allele) formats. Haploview defines 3 haplotype blocks: Block 1 (S1-S2-S3-S4), Block 2 (S5-S6-S7-S8-S9) and Block 3 (S10-S11), where S1 = rs5761635, S2 = rs2283843, S3 = rs5997109, S4 = rs2071860, S5 = rs2071861, S6 = rs2071862, S7 = rs4276, S7 = rs2239832, S8 = rs5752359, S9 = rs2009066, S10 = rs1018833, and S11 = rs739310. For details, see Figure 1. For haplotype analysis by Plink, this table shows the *best sliding window only*; the best sliding window consists of two SNPs (S8 = rs5752359, S9 = rs2009066). SNPs S8 and S9 are underlined for the sake of easy cross referencing between Haploview-defined block 2 and Plink’s best sliding window.

† Nominal *P* value is indicated as *P*_nom_ while empirical p value generated by 10,000 permutations is indicated as *P*_emp_.

For the replication sample set and as defined by confidence bounds [20], LD blocks for the combined group of cases and controls were the same as those for the controls only (Figure 1). However, they were slightly different from those for the cases only. There were also three LD blocks for the Han Chinese subjects of the HapMap database, but the second and the third blocks were very different those for the combined group of cases and controls (Figure 1). In general, the LD among SNPs was slightly stronger in the Han Chinese of the HapMap database than in the Chinese subjects of the replication sample set. For the discovery sample set, three *CRYBA4* SNPs had been genotyped (Table 3) and the LD measures among them (data not shown) were almost identical to their corresponding counterparts in the replication sample set.

### Meta-analysis of rs2071861, rs2239832 and rs2009066

Because rs2071861, rs2239832 and rs2009066 of CRYBA4 were the most significantly associated SNPs (Table 4), meta-analysis was used to summarize their overall significance (Table 6). Of these, rs2009066 displayed the highest degree of significance. Under the allelic genetic model, there was no significant heterogeneity (P = 0.259) between the odds ratios (ORs) from the two sample sets, and overall significant association with high myopia could be detected (P = 1.54e-5 and OR (95% CI) = 1.41 (1.21–1.64)). Similarly, under the dominant genetic model, no significant heterogeneity (P = 0.063) could be detected across the two sample sets, and significant association was demonstrated in the combined analysis (P = 1.73e-5 and OR (95% CI) = 1.74 (1.35–2.25)). Similar results were also obtained for rs2071861 and rs2239832 (Table 6). Overall results from meta-analysis did match with the findings from individual studies that highly significant association could be detected. This combined analysis confirmed that polymorphisms of CRYBA4 were significantly associated with high myopia.

**Table 6:** Meta-analysis of three *CRYBA4* SNPs.

SNP		Allelic model (alleles 2 vs 1)*			Dominant model (genotypes 22+12 vs 11)*		
(allele 2, allele 1)	Study	Exact test,*P*	OR (95% CI)	Breslow-Daytest, *P* †	Exact test,p	OR (95% CI)	Breslow-Day test, *P* †
**rs2071861**	Initial	0.019	1.30 (1.04–1.61)	–	0.077	1.37 (0.97–1.95)	–
(A, G)	Replication	0.007	1.36 (1.09–1.70)	–	3.10e-04	2.03 (1.38–2.97)	–
	Combined †	3.43e-04	1.33 (1.14–1.55)	0.765	1.50e-04	1.64 (1.27–2.12)	0.141
							
**rs2239832**	Initial ‡	0.020	1.29 (1.05–1.60)	–	0.077	1.39 (0.98–1.97)	–
(A, G)	Replication	0.006	1.37 (1.10–1.71)	–	7.99e-05	2.13 (1.46–3.12)	–
	Combined †	2.78e-04	1.33 (1.14–1.55)	0.713	5.37e-05	1.69 (1.31–2.19)	0.102
							
**rs2009066**	Initial ‡	0.020	1.29 (1.05–1.60)	–	0.077	1.39 (0.98–1.97)	–
(G, A)	Replication	1.88e-04	1.55 (1.23–1.94)	–	2.04e-05	2.25 (1.55–3.27)	–
	Combined †	1.54e-05	1.41 (1.21–1.64)	0.259	1.73e-05	1.74 (1.35–2.25)	0.063

* Allele 1 is the reference allele for the allelic model while genotype 11 is the reference genotype for the dominant model.

† The Breslow-Day test tests the null hypothesis of homogeneity of the odds ratios across the initial and the replication studies. The combined study combines the data from the initial study (342 cases and 342 controls) and the replication study (316 cases and 313 controls) by means of Mantel-Haenszel test.

‡ Based on genotype data imputed using the Beagle package with the genotype data from the replication study as the reference panel.

OR, odds ratio; CI, confidence intervals.

## Discussion

The present case-control study identified a novel susceptibility gene (*CRYBA4*) for high myopia in southern Chinese. Existing linkage evidences [12]–[16] strongly suggest that the *MYP6* locus is very likely to harbour a predisposing gene for myopia. We performed a systematic genetic association study using southern Chinese subjects in Hong Kong. Within a case-control study framework, subjects were recruited with stringent criteria in the extremes of the visual spectrum. It is believed that, with extreme phenotypic contrast (high myopes as cases and emmetropes as controls), the case and the control groups would be enriched with subjects with and without genetic predisposing factors respectively so that environmental factors would have minimal effect, i.e., testing association with genetically-determined myopia. Therefore, using such sample sets for testing genetic association will achieve better statistical power and hence higher chance to detect a susceptibility gene if there is one. Power calculation by Quanto (version 1.24) [21] shows that the *replication sample set* achieved ≥80% statistical power for a dominant model at α = 0.002 ( = 0.05/25 for 25 SNPs genotyped in the replication stage) under the following scenarios: risk allele frequency of 0.125 to 0.425 for OR = 2.00, and risk allele frequency of 0.075 to 0.50 for OR = 2.25 (ORs taken from Table 6). Similar results were also obtained for the *combined sample set*: ≥80% power for a dominant model at α = 0.002 when the risk allele frequency ranges from 0.120 to 0.450 for OR = 1.65 or when the risk allele frequency is between 0.080 and 0.500 for OR = 1.75 (ORs taken from Table 6).

With well-defined case-control subjects, our study used a positional candidate gene approach to mapping the myopia susceptibility gene in the *MYP6* interval. In addition to the traditional subjective candidate gene identification based on published literature and biological relevance, objective *in silico* prioritization of candidates in the *MYP6* locus was also done. With independent support from the objective counterpart, our candidate genes were selected with strong justification. This provided us with more confidence to carry on the subsequent steps. Indeed, by the initial and the replication case-control association studies, we found significant association of *CRYBA4* with high myopia. Meta-analysis of the combined data further confirmed the findings. We also analysed the genotype data with adjustment for sex, age and batch effect of sample sets (as covariates in logistic regression) to account for their potential confounding effects, and the original significant association for *CRYBA4* (with rs2009066 showing the strongest significance) remained unchanged (results not shown). In other words, the positive association between *CRYBA4* and high myopia is robust to these potential confounding factors. To our knowledge, this is the first report of *CRYBA4* being a myopia susceptibility gene.

We have used exact test to test whether genotypes in controls were in HWE or not, and a *P*<0.001 was adopted as the threshold for excluding SNPs from analysis due to violation of HWE. The exact test for HWE is conservative at an α level of 0.001 [22], which is also used in many large-scale association studies (e.g. [23]). Some of the associated SNPs showed a *P* value above 0.001, but below 0.05, for HWE testing (Table 3). An alpha level of 0.05 is even more widely used as the threshold to define HWE. This might raise a minor concern in the interpretation of the results. Therefore, we recommend that our findings be replicated by other researcher groups with more independent sample sets. It is also noteworthy that some SNPs showed very significant differences in genotype distribution between cases and controls, and gave unexpectedly very low *P* values in the initial study (e.g. *P*  = 4.86e-19 for rs2800960, and *P* = 4.86e-106 for rs4616572; Table 3). These were very likely the results of genotyping case and control samples on separate plates – the so-called “batch” effects, which might not be distinguishable from “phenotype status” effects. Therefore, it is advisable to have equal numbers of case and control samples on each sample plate so as to avoid possible batch effects. More importantly, replication study by an independent sample set is crucial. A third noteworthy point is about LD patterns in the *CRYBA4* locus. The LD among SNPs was slightly stronger in HapMap Han Chinese subjects (n = 45) than in the Chinese subjects of the replication set (n = 629). This variation in the LD measures give rise to different boundaries between two LD blocks (Blocks 2 and 3; Figure 1) for these two Chinese populations. This fine-scale variation in LD patterns is not uncommon because LD is influenced by population histories among other things, as has also been observed in some of our previous studies [24]–[26].

Crystallins are water-soluble proteins and are major structural components in the lens of the eyes (constituting 80–90% of the soluble protein fraction). In human lens, crystallins are divided into three families: α-crystallin, β-crystallin and γ-crystallin, which account for 40%, 35% and 25% of the total crystallin protein respectively [27]. Their stability and proper interactions are important for transparency and refractive index of the lens. In particular, β-crystallin family consists of three basic (CRYBB1-3) and four acidic (CRYBA1-4) protein members. The *CRYBA4* gene encodes the βA4-crystallin chain of 196 amino acid residues, which makes up ∼5% of the total soluble protein in young human lens [28]. In the present study, we identified rs2009066 to be the SNP most significantly associated with high myopia, and this SNP is located 3 kb downstream of *CRYBA4*. Based on an online tool for SNP function prediction (SNPinfo; http://manticore.niehs.nih.gov/snpfunc.htm), rs2009066 does not seem to have any predicted functional role. Moreover, the criteria for tag SNP selection (r^2^ cut-off of 0.8 and minor allele frequency cut-off of 0.1) might not adequately capture all the sequence variants into consideration. Therefore, the association is likely to be driven by an untyped causal variant in LD with rs2009066 or other associated SNPs although the possibility of some undiscovered functional roles for the associated SNPs could not be ruled out entirely. It is worth undertaking re-sequencing of the *CRYBA4* gene and flanking regions for diseased subjects to discover any potentially functional target. It is also useful to investigate more sequence variants (both functional and non-functional) in other ethnic groups to improve coverage of the gene and to be used for comparison.

Current literature provides indirect support for our findings. First, previous studies have identified mutations in the *CRYBA4* gene responsible for cataract, microcornea and microphthalmia [27], [29]. There are reports of these ocular abnormalities found together with myopia [30]–[33]. These ocular disorders may partly share their underlying pathology, which supports the present finding of association between *CRYBA4* and high myopia. Second, although there is no report of *CRYBA4* expressions in locations other than the lens in humans, animal studies have identified *CRYBA4* expression in the retina and sclera [34]–[37]. As most high myopia cases are of axial type (excessive elongation of eyeballs), βA4-crystallin, or in interaction with other crystallin members, may have a role in leading to axial change caused by some processes outside the lens. Last but not least, crystallins, including βA4-crystallin, may have a more versatile role than just a lens constituent. Studies have already highlighted the potential significance of crystallins in stress response [38]–[40]. *CRYBA4* and many other crystallin genes show strong and sustained up-regulation after retinal injury [33], [34], and expression changes in both protein and mRNA levels in the sclera of guinea pig during *form deprivation myopia* and subsequent recovery [37]. These findings suggest their potentially important roles in retinal wound healing process and stress response, perhaps in retinal and sclera remodelling as well. In addition, previous animal studies have also reported the regulation of *CRYBA4* or other crystallin genes by transcription factors such as Pax6 and Maf. The transcription factor PAX6 could repress the expression of lens fibre cell-specific *CRYBB1* gene expression in chicken and mouse [41], [42], suggestively through blocking the Maf-mediated transactivation of *CRYBB1* promoter [43]. Apart from regulating *CRYBB1*, mouse recombinant Maf could also bind to the promoters of some crystallin genes including *CRYBA4* [44]. This indicates that Maf might directly activate many crystallin genes. Moreover, another mouse study suggested that tissue-specific over-expression of Rybp (a zinc finger protein) in the lens could reduce *CRYBA4* gene expression while heterozygous Rybp null mice often resulted in retinal coloboma characterized by expanding localization of PAX6 [45]. PAX6 has a central role in eye development [46] and has also been shown to be associated with high myopia [24], [25], [47]. Since crystallins have been suggested to be in close relationship with PAX6 as well as other interacting transcription factors and proteins, genetic variants in *CRYBA4* may lead to myopic change or other ocular symptoms through disrupted regulatory network in eye development. Indeed, βA4-crystallin was found to interact with βB1-crystallin [48] and βB2-crystallin monomers [49]. Perhaps, the impact of *CRYBA4* might exhibit via synergistic effect with other crystallin members. This may shed light on the potential gene-gene interaction network in myopia aetiology. Although environmental influence may be relatively small, their potential interaction with genetic factors could complicate the situation to a certain extent.

In conclusion, with the findings from the initial and replication studies as well as summary data from meta-analysis, we discovered significant association between *CRYBA4* and high myopia for the first time. Furthermore, our study signified the potential importance of appropriate gene prioritization (manual and *in silico*) in candidate selection. This analysis would add important value and confidence to the subsequent steps in disease gene mapping pipeline.

## Materials and Methods

### Subject Recruitment

Unrelated southern Chinese subjects in Hong Kong were recruited for this study. They were recruited through the use of promotion posters put up throughout the campus of the university, through the use of visual screening activities outside the campus, and through referrals of myopic individuals from local optometrists. The entry criteria were spherical equivalent (SE) of -8.00 D or worse for both eyes for cases with high myopia, and SE within ±1.00 D for both eyes for emmetropic controls. Subjects were excluded if they showed obvious signs of ocular disease or other inherited disease associated with myopia. Written informed consent was obtained from all subjects. The study obtained ethics approval from the Human Subjects Ethics Subcommittee of the Hong Kong Polytechnic University, and adhered to the tenets of the Declaration of Helsinki. Details of ocular examination, blood collection and DNA extraction have been reported previously [50].

### Candidate Gene Selection and Subsequent Validation by Endeavour

Genes were retrieved from the *MYP6* locus (22q11.2-q13.2; 17900001-44200000, NCBI build 17.1), and manually prioritized into five categories (in decreasing relevance): highly relevant genes, possibly related genes, unlikely genes, homologous genes, and pseudo, putative or hypothetical genes. This subjective prioritization was supported by extensive literature via manual search. In addition, the genes retrieved were also prioritized objectively with an *in silico* prioritization tool Endeavour [17]. Details and supporting literature are provided in the online Appendix S1. Genes prioritized with these two methods were compared. Genes in the “highly relevant” category were selected for study.

### SNP Selection

For the initial study, tag SNPs were selected from candidate genes in the “highly relevant” category. The genomic regions of interest included the gene loci selected and their respective flanking regions (3 kb upstream and 3 kb downstream). The selection was based on the Han Chinese data (release #24, phase II) of the HapMap Project [18], [19] through the HapMap’s Tagger software interface using multimarker tagging method with r^2^ cut-off of 0.8 and minor allele frequency cut-off of 0.1.

For the replication study, candidate genes to be followed up were chosen based on the set-based association results of the initial study. SNPs were then selected from the corresponding candidate genes on the basis of the linkage disequilibrium (LD) pattern and functional relevance (located at predicted transcription factor or microRNA binding sites by SNPinfo (http://manticore.niehs.nih.gov/snpfunc.htm).

### SNP Genotyping

Genotyping was done using the MassARRAY iPLEX GOLD platform (Sequenom, San Diego, CA) at the Genome Research Centre of a local university (http://genome.hku.hk/portal/) as a contracted service. The manufacturer’s protocols were followed closely. Genotypes were called after cluster analysis using the default setting of Gaussian mixture model. Genotype calls were then further reviewed manually to undo any uncertain calls due to clustering artifact. Assay with less than 80% call rate within the same SpectroChip was considered failed. For every 96-well sample plate, one well was used for blank control and five wells for duplicate check. SpectroChip with more than 25% call rate in the blank control was considered failed and would be repeated. SpectroChip with less than 99.5% concordance in duplicate checks along with more than 10% call rate in blank check was also considered failed.

### Imputation of Genotypes for rs2239832 and rs2009066 in the Discovery Sample Set

Genotypes of rs2239832 and rs2009066 were imputed by Beagle [51] for the discovery sample set, which had *not* been genotyped for these two SNPs in the initial study. Genotype data from the replication study were used as the reference panel.

### Statistical Analysis

Ocular data were analysed by SPSS (v16.0) (Chicago, IL). Genotypes were tested for Hardy-Weinberg equilibrium (HWE) in control subjects by exact test [22] implemented in PLINK [52], and SNPs with *P* value <0.001 were discarded to avoid potential genotyping errors. Genetic association was tested using Fisher’s exact tests under allelic, dominant and recessive models by PLINK (v1.07) [52]. Correction for multiple comparisons was performed by permutation (swapping of the case-control status). In each round of permutation, the best *original* result of every SNP was compared against the best result of the three tests (allelic, dominant and recessive) of that SNP, and also against the best results from all SNPs. Set-based test implemented in PLINK was used to estimate empirical significance of individual candidate genes with parameters *P* = 0.05 and r^2^ = 0.8. Haplotypes were defined by variable-sized sliding windows or haplotype blocks, and the corresponding haplotype association tests were performed by PLINK or Haploview (v4.2) [53] respectively. LD pattern of the *CRYBA4* gene was generated by Haploview. Multiple testing was corrected by permutations to assess the empirical significance (see table footnotes for details). Meta-analysis was performed using the fixed-effect Mantel-Haenszel model to summarize the association results from the discovery and the replication sample sets, and Breslow-Day test was used to test for heterogeneity in odds ratios.

## Supporting Information

Table S1 Single marker analysis of 178 SNPs (from 26 genes) by PLINK for the discovery sample set.
(DOC)

Appendix S1 Candidate gene selection from the *MYP6* locus and subsequent validation by Endeavour.
(DOC)
