# Supplementary material for: Association of High Myopia with Crystallin Beta A4 (CRYBA4) Gene Polymorphisms in the Linkage-Identified MYP6 Locus
Source: PLoS One. 2012 Jun 29;7(6):e40238. doi: 10.1371/journal.pone.0040238 (PMC3389832; doi:10.1371/journal.pone.0040238)
Supplement: Table S1 — Single marker analysis of 178 SNPs (from 26 genes) by PLINK for the discovery sample set. (DOC) [file pone.0040238.s001.doc]

**Table S1.** Single marker analysis of 178 SNPs (from 26 genes) by PLINK for the discovery sample set

| SNP | Physical position on chr 22 (bp) † | Allele * | | Genotype (22/12/11) | | P value for HWE testing in controls | Fisher's exact test *P* | | | *P*_emp_ ‡ |
| --- | --- | --- | --- | --- | --- | --- | --- | --- | --- | --- |
|  |  | 2 | 1 | Case | Control |  | Allelic | Dominant | Recessive |  |
| ***PEX26*** |  |  |  |  |  |  |  |  |  |  |
| rs464541 | 18562888 | T | C | 36/147/155 | 35/150/154 | 0.902 | 1.000 | 0.939 | 0.901 | 1.000 |
| rs362262 | 18565048 | T | C | 28/129/183 | 30/128/182 | 0.279 | 0.903 | 1.000 | 0.891 | 1.000 |
| rs17207360 | 18566917 | T | C | 11/109/219 | 14/98/227 | 0.375 | 0.782 | 0.571 | 0.684 | 1.000 |
|  |  |  |  |  |  |  |  |  |  |  |
| ***DGCR6*** |  |  |  |  |  |  |  |  |  |  |
| rs372579 | 18893238 | A | G | 7/87/248 | 9/99/234 | 0.849 | 0.268 | 0.276 | 0.801 | 1.000 |
| rs3810600 | 18893248 | C | T | 16/125/197 | 20/133/182 | 0.570 | 0.282 | 0.313 | 0.498 | 1.000 |
| rs5993409 | 18894058 | G | C | 5/82/248 | 12/84/240 | 0.221 | 0.250 | 0.488 | 0.138 | 1.000 |
| rs5746636 | 18896301 | G | T | 42/166/127 | 63/160/114 | 0.655 | 0.058 | 0.296 | **0.033** | 0.999 |
| rs3890992 | 18896605 | A | G | 0/65/277 | 0/74/268 | 0.021 | 0.474 | 0.447 | 1.000 | 1.000 |
| rs372055 | 18900750 | G | A | 6/78/253 | 9/82/247 | 0.515 | 0.481 | 0.598 | 0.603 | 1.000 |
| rs395440 | 18901878 | C | G | 3/61/272 | 4/66/263 | 1.000 | 0.533 | 0.563 | 0.724 | 1.000 |
|  |  |  |  |  |  |  |  |  |  |  |
| ***DGCR5*** |  |  |  |  |  |  |  |  |  |  |
| rs2077200 | 18956203 | G | A | 21/109/203 | 18/123/195 | 0.881 | 0.698 | 0.479 | 0.624 | 1.000 |
| rs9680846 | 18965418 | T | C | 22/95/216 | 18/101/217 | 0.179 | 0.840 | 1.000 | 0.518 | 1.000 |
| rs11912325 | 18966001 | A | C | 43/130/160 | 36/154/149 | 0.716 | 0.728 | 0.314 | 0.402 | 1.000 |
| rs2540651 | 18972331 | A | G | 40/148/145 | 37/150/153 | 1.000 | 0.644 | 0.756 | 0.717 | 1.000 |
| rs2075198 | 18979268 | C | T | 2/50/284 | 2/56/281 | 1.000 | 0.625 | 0.603 | 1.000 | 1.000 |
| rs2075197 | 18980239 | A | G | 4/36/292 | 3/63/273 | 1.000 | **0.023** | **0.011** | 0.723 | 0.917 |
| rs1210637 | 18980443 | T | C | 45/153/136 | 41/160/138 | 0.638 | 0.820 | 1.000 | 0.645 | 1.000 |
|  |  |  |  |  |  |  |  |  |  |  |
| ***DGCR2*** |  |  |  |  |  |  |  |  |  |  |
| rs2073776 | 19024651 | A | G | 36/141/161 | 34/141/166 | 0.611 | 0.769 | 0.818 | 0.802 | 1.000 |
| rs2000996 | 19034829 | G | A | 49/165/118 | 53/167/114 | 0.572 | 0.655 | 0.745 | 0.747 | 1.000 |
| rs17810649 | 19035072 | G | A | 4/87/249 | 4/74/263 | 0.801 | 0.296 | 0.250 | 1.000 | 1.000 |
| rs2238743 | 19049602 | G | T | 50/167/120 | 53/169/118 | 0.652 | 0.782 | 0.810 | 0.831 | 1.000 |
| rs2238754 | 19058146 | T | C | 20/126/191 | 16/122/202 | 0.758 | 0.406 | 0.484 | 0.498 | 1.000 |
| rs2800960 | 19077049 | T | C | 0/55/265 | 0/0/337 | 1.000 | **1.89e-18** | **4.86e-19** | 1.000 | **1.00e-06** |
| rs2800969 | 19077594 | T | C | 14/103/219 | 16/122/201 | 0.757 | 0.161 | 0.131 | 0.852 | 1.000 |
| rs1934895 | 19080150 | C | T | 8/66/267 | 4/78/259 | 0.627 | 0.805 | 0.523 | 0.383 | 1.000 |
| rs738905 | 19097837 | A | T | 3/39/299 | 2/36/303 | 0.320 | 0.654 | 0.721 | 1.000 | 1.000 |
| rs807740 | 19102582 | C | G | 26/131/180 | 19/131/189 | 0.663 | 0.353 | 0.589 | 0.284 | 1.000 |
| rs807741 | 19103298 | T | G | 3/81/257 | 6/80/253 | 1.000 | 0.689 | 0.860 | 0.339 | 1.000 |
| rs807744 | 19104186 | T | A | 37/146/151 | 27/155/156 | 0.204 | 0.447 | 0.817 | 0.190 | 1.000 |
|  |  |  |  |  |  |  |  |  |  |  |
| ***DGCR14*** |  |  |  |  |  |  |  |  |  |  |
| rs1052773 | 19119938 | A | G | 7/85/248 | 6/78/257 | 1.000 | 0.481 | 0.485 | 0.788 | 1.000 |
| rs2285858 | 19120959 | A | C | 15/120/206 | 11/115/214 | 0.403 | 0.426 | 0.529 | 0.549 | 1.000 |
| rs16983371 | 19123952 | C | T | 19/117/199 | 20/126/194 | 1.000 | 0.609 | 0.585 | 1.000 | 1.000 |
| rs5747997 | 19124375 | C | T | 54/164/121 | 57/172/109 | 0.504 | 0.440 | 0.372 | 0.757 | 1.000 |
| rs5747998 | 19124546 | C | G | 6/57/278 | 4/66/269 | 1.000 | 0.659 | 0.499 | 0.752 | 1.000 |
| rs737923 | 19132325 | A | G | 34/129/172 | 33/136/171 | 0.437 | 0.905 | 0.818 | 0.898 | 1.000 |
| rs715544 | 19133605 | A | G | 18/98/222 | 16/113/210 | 0.872 | 0.502 | 0.337 | 0.729 | 1.000 |
| rs4819776 | 19133916 | T | C | 48/167/122 | 46/166/128 | 0.565 | 0.696 | 0.750 | 0.825 | 1.000 |
|  |  |  |  |  |  |  |  |  |  |  |
| ***GP1BB*** |  |  |  |  |  |  |  |  |  |  |
| rs3810596 | 19710461 | G | C | 0/8/332 | 0/1/341 | 1.000 | **0.021** | **0.020** | 1.000 | 0.990 |
|  |  |  |  |  |  |  |  |  |  |  |
| ***ARVCF*** |  |  |  |  |  |  |  |  |  |  |
| rs4646316 § | 19952132 | T | C | 45/111/157 | 60/86/136 | **1.07e-08** | - | - | - | - |
| rs165774 | 19952561 | A | G | 5/87/248 | 3/82/253 | 0.236 | 0.527 | 0.600 | 0.725 | 1.000 |
| rs174696 | 19953176 | T | C | 88/142/102 | 53/176/109 | 0.219 | **0.024** | 0.678 | **0.001** | 0.152 |
| rs174697 | 19953832 | A | G | 48/158/130 | 42/165/127 | 0.350 | 0.866 | 0.874 | 0.571 | 1.000 |
| rs165815 | 19959473 | T | C | 78/183/79 | 76/175/86 | 0.514 | 0.625 | 0.531 | 0.927 | 1.000 |
| rs2518823 § | 19960188 | C | T | 94/129/114 | 85/118/129 | **4.48e-07** | - | - | - | - |
| rs12158201 | 19966749 | T | C | 4/38/296 | 0/48/293 | 0.392 | 0.915 | 0.572 | 0.061 | 1.000 |
| rs1990277 | 19970502 | A | G | 47/161/129 | 35/150/155 | 1.000 | **0.035** | 0.062 | 0.158 | 1.000 |
| rs2238781 | 19970547 | T | G | 12/104/223 | 16/130/195 | 0.373 | **0.029** | **0.022** | 0.563 | 0.993 |
| rs917478 | 19972824 | C | T | 13/102/223 | 16/129/195 | 0.453 | **0.034** | **0.022** | 0.705 | 0.993 |
| rs2238786 | 19975444 | G | A | 5/74/261 | 9/96/236 | 1.000 | **0.026** | **0.031** | 0.419 | 0.997 |
| rs756653 | 19976845 | T | C | 3/77/257 | 9/96/235 | 1.000 | **0.021** | **0.039** | 0.143 | 0.991 |
| rs2012714 | 19977647 | A | G | 5/82/250 | 11/109/221 | 0.727 | **0.007** | **0.010** | 0.205 | 0.792 |
| rs2238791 § | 19983111 | A | C | 61/170/107 | 15/188/137 | **4.33e-07** | - | - | - | - |
| rs17817568 | 19983932 | T | C | 3/45/284 | 0/41/288 | 0.624 | 0.331 | 0.495 | 0.249 | 1.000 |
| rs2073741 | 19989890 | A | G | 2/59/281 | 3/83/255 | 0.236 | **0.025** | **0.020** | 0.686 | 0.988 |
| rs4819853 § | 19999760 | A | G | 0/318/24 | 0/93/209 | **2.90e-04** | - | - | - | - |
| rs3788322 | 20006040 | T | C | 52/161/125 | 38/149/150 | 0.903 | **0.027** | 0.050 | 0.141 | 0.998 |
| rs1858770 | 20006992 | C | A | 2/51/286 | 3/83/255 | 0.236 | **0.004** | **0.002** | 1.000 | 0.435 |
|  |  |  |  |  |  |  |  |  |  |  |
| ***MMP11*** |  |  |  |  |  |  |  |  |  |  |
| rs131451 | 24113544 | C | T | 71/164/104 | 65/165/109 | 0.912 | 0.585 | 0.741 | 0.632 | 1.000 |
| rs738790 | 24116817 | T | C | 28/122/189 | 18/133/189 | 0.468 | 0.576 | 1.000 | 0.130 | 1.000 |
| rs738791 | 24117525 | T | C | 43/127/157 | 41/153/140 | 1.000 | 0.324 | 0.119 | 0.816 | 1.000 |
|  |  |  |  |  |  |  |  |  |  |  |
| ***GSTT1*** |  |  |  |  |  |  |  |  |  |  |
| rs6004035 § | 24385247 | - | - | **0/0/0** | **0/0/0** | 1.000 | - | - | - | - |
|  |  |  |  |  |  |  |  |  |  |  |
| *ADORA2A* |  |  |  |  |  |  |  |  |  |  |
| rs2267076 | 24830595 | T | C | 68/170/98 | 67/167/105 | 1.000 | 0.702 | 0.616 | 0.923 | 1.000 |
| rs8184997 | 24833732 | T | C | 1/53/285 | 3/53/284 | 0.729 | 0.769 | 0.917 | 0.624 | 1.000 |
| rs2236624 | 24836024 | T | C | 52/169/119 | 52/168/121 | 0.653 | 0.956 | 0.936 | 1.000 | 1.000 |
| rs5760423 | 24840118 | G | T | 73/174/90 | 75/171/94 | 0.914 | 0.957 | 0.796 | 0.926 | 1.000 |
| rs5751878 | 24841990 | T | C | 1/53/287 | 3/52/286 | 0.722 | 0.844 | 1.000 | 0.624 | 1.000 |
|  |  |  |  |  |  |  |  |  |  |  |
| ***CRYBB3*** |  |  |  |  |  |  |  |  |  |  |
| rs2001159 | 25596870 | T | C | 60/167/106 | 45/173/121 | 0.208 | 0.120 | 0.328 | 0.111 | 1.000 |
| rs2269672 | 25597331 | T | C | 35/145/161 | 24/149/168 | 0.292 | 0.316 | 0.646 | 0.173 | 1.000 |
| rs5752079 | 25597842 | G | A | 6/62/273 | 6/74/260 | 0.805 | 0.314 | 0.266 | 1.000 | 1.000 |
| rs732706 | 25600699 | A | G | 86/162/87 | 74/179/87 | 0.385 | 0.549 | 0.930 | 0.241 | 1.000 |
| rs2252878 | 25602708 | G | A | 40/156/144 | 26/162/153 | 0.078 | 0.205 | 0.537 | 0.071 | 1.000 |
| rs5760907 | 25604477 | G | A | 57/169/116 | 49/167/122 | 0.569 | 0.439 | 0.574 | 0.460 | 1.000 |
| rs5760908 | 25604716 | T | G | 3/59/270 | 1/56/282 | 0.491 | 0.450 | 0.546 | 0.369 | 1.000 |
|  |  |  |  |  |  |  |  |  |  |  |
| ***CRYBB2*** |  |  |  |  |  |  |  |  |  |  |
| rs4822578 | 25613103 | A | G | 16/113/212 | 5/112/218 | 0.027 | 0.172 | 0.472 | **0.024** | 0.996 |
| rs2267077 | 25616065 | G | C | 44/166/127 | 36/166/134 | 0.155 | 0.396 | 0.580 | 0.405 | 1.000 |
| rs969623 | 25616213 | C | T | 28/150/161 | 34/143/162 | 0.800 | 0.814 | 1.000 | 0.506 | 1.000 |
| rs16979774 | 25617346 | T | C | 9/89/241 | 4/88/249 | 0.269 | 0.403 | 0.608 | 0.174 | 1.000 |
| rs739315 | 25617602 | G | A | 68/166/107 | 55/173/110 | 0.373 | 0.381 | 0.805 | 0.232 | 1.000 |
| rs5752083 | 25618562 | G | T | 19/118/205 | 20/140/182 | 0.333 | 0.149 | 0.090 | 1.000 | 1.000 |
| rs8142586 | 25619267 | A | G | 6/80/252 | 8/93/237 | 1.000 | 0.221 | 0.229 | 0.788 | 1.000 |
| rs6004490 | 25619339 | C | T | 12/123/203 | 16/136/189 | 0.192 | 0.222 | 0.244 | 0.564 | 1.000 |
| rs5752084 | 25621591 | C | T | 2/68/269 | 5/68/267 | 0.788 | 0.665 | 0.851 | 0.451 | 1.000 |
|  |  |  |  |  |  |  |  |  |  |  |
| ***HPS4*** |  |  |  |  |  |  |  |  |  |  |
| rs929131 | 26844769 | T | C | 20/98/219 | 17/96/224 | 0.117 | 0.633 | 0.746 | 0.736 | 1.000 |
| rs3747129 | 26862041 | A | G | 19/130/192 | 12/128/198 | 0.159 | 0.371 | 0.587 | 0.270 | 1.000 |
| rs1023495 | 26865054 | A | G | 13/83/242 | 11/87/243 | 0.320 | 1.000 | 0.933 | 0.684 | 1.000 |
| rs5997095 | 26865973 | A | G | 30/140/171 | 20/158/163 | 0.025 | 0.953 | 0.592 | 0.186 | 1.000 |
| rs5761552 | 26870502 | T | C | 93/152/93 | 71/190/79 | 0.039 | 0.704 | 0.217 | **0.049** | 1.000 |
| rs9608491 | 26870985 | G | A | 4/65/267 | 2/48/292 | 1.000 | **0.039** | **0.044** | 0.447 | 1.000 |
|  |  |  |  |  |  |  |  |  |  |  |
| ***CRYBB1*** |  |  |  |  |  |  |  |  |  |  |
| rs5761618 | 26993706 | G | T | 82/173/84 | 84/164/92 | 0.517 | 0.786 | 0.540 | 0.929 | 1.000 |
| rs2301440 | 26997482 | T | C | 26/119/193 | 23/122/196 | 0.470 | 0.802 | 0.938 | 0.659 | 1.000 |
| rs9613228 § | 27002223 | A | G | **0/5/1** | **0/6/0** | 0.091 | - | - | - | - |
| rs5752354 | 27002648 | C | T | 7/102/230 | 8/91/242 | 1.000 | 0.510 | 0.406 | 1.000 | 1.000 |
| rs5761627 | 27004298 | G | A | 58/148/128 | 66/162/112 | 0.582 | 0.167 | 0.149 | 0.551 | 1.000 |
| rs5761631 | 27007269 | A | G | 28/127/182 | 27/128/185 | 0.489 | 0.903 | 0.939 | 0.889 | 1.000 |
|  |  |  |  |  |  |  |  |  |  |  |
| ***CRYBA4*** |  |  |  |  |  |  |  |  |  |  |
| rs5761635 | 27014467 | T | C | 48/172/121 | 38/164/137 | 0.342 | 0.145 | 0.206 | 0.299 | 1.000 |
| rs5997109 | 27017435 | G | C | 83/166/90 | 78/175/86 | 0.588 | 1.000 | 0.793 | 0.718 | 1.000 |
| rs2071861 | 27021189 | A | G | 93/169/75 | 70/174/96 | 0.663 | **0.019** | 0.077 | **0.039** | 0.986 |
|  |  |  |  |  |  |  |  |  |  |  |
|  |  |  |  |  |  |  |  |  |  |  |
| ***XBP1*** |  |  |  |  |  |  |  |  |  |  |
| rs2267131 § | 29190471 | C | T | 13/123/202 | 33/74/224 | **2.30e-08** | - | - | - | - |
| rs2269577 | 29196757 | G | C | 33/142/160 | 38/139/161 | 0.380 | 0.769 | 1.000 | 0.616 | 1.000 |
|  |  |  |  |  |  |  |  |  |  |  |
| ***NF2*** |  |  |  |  |  |  |  |  |  |  |
| rs9613999 | 29997011 | T | C | 7/96/235 | 8/104/229 | 0.454 | 0.563 | 0.511 | 1.000 | 1.000 |
| rs9614005 § | 30002603 | G | T | 24/82/228 | 25/89/221 | **7.58e-04** | - | - | - | - |
| rs2531844 | 30007808 | T | C | 14/121/203 | 20/121/195 | 0.881 | 0.437 | 0.638 | 0.297 | 1.000 |
| rs2530664 | 30038152 | A | C | 63/157/118 | 68/164/108 | 0.741 | 0.411 | 0.415 | 0.698 | 1.000 |
| rs6006219 | 30049643 | T | C | 2/45/292 | 4/43/294 | 0.105 | 0.917 | 1.000 | 0.686 | 1.000 |
| rs2252587 | 30064731 | G | A | 52/169/120 | 52/175/113 | 0.264 | 0.741 | 0.628 | 1.000 | 1.000 |
| rs2527335 | 30065967 | G | T | 10/97/230 | 16/118/207 | 1.000 | **0.034** | **0.045** | 0.318 | 1.000 |
| rs2530678 | 30081715 | A | G | 74/147/117 | 59/164/117 | 0.911 | 0.442 | 1.000 | 0.147 | 1.000 |
| rs1009148 | 30088442 | C | T | 48/157/128 | 42/158/138 | 0.813 | 0.428 | 0.529 | 0.497 | 1.000 |
| rs2267152 | 30088671 | C | T | 26/127/185 | 24/133/182 | 1.000 | 0.951 | 0.817 | 0.771 | 1.000 |
| rs2530681 | 30097039 | C | G | 60/150/129 | 63/164/109 | 0.912 | 0.224 | 0.147 | 0.765 | 1.000 |
|  |  |  |  |  |  |  |  |  |  |  |
| ***OSM*** |  |  |  |  |  |  |  |  |  |  |
| rs8138196 | 30656635 | G | A | 28/154/145 | 30/148/157 | 0.611 | 0.679 | 0.533 | 0.891 | 1.000 |
| rs7289095 | 30658722 | T | C | 19/118/205 | 20/106/215 | 0.151 | 0.558 | 0.432 | 0.871 | 1.000 |
| rs1476576 | 30660517 | A | C | 26/123/192 | 19/148/172 | 0.101 | 0.462 | 0.166 | 0.355 | 1.000 |
| rs10854598 | 30664920 | A | G | 41/157/142 | 46/132/159 | 0.036 | 0.492 | 0.164 | 0.567 | 1.000 |
|  |  |  |  |  |  |  |  |  |  |  |
| ***SMTN*** |  |  |  |  |  |  |  |  |  |  |
| rs12157641 | 31481922 | C | G | 76/170/91 | 65/158/116 | 0.436 | 0.056 | **0.045** | 0.298 | 1.000 |
| rs2074736 | 31492482 | G | A | 77/163/99 | 92/168/79 | 0.914 | 0.065 | 0.097 | 0.214 | 1.000 |
| rs10304 | 31500536 | A | G | 55/168/115 | 69/166/103 | 0.913 | 0.170 | 0.365 | 0.196 | 1.000 |
|  |  |  |  |  |  |  |  |  |  |  |
| ***TIMP3*** |  |  |  |  |  |  |  |  |  |  |
| rs1962223 | 33193905 | C | G | 39/162/133 | 37/159/140 | 0.470 | 0.648 | 0.638 | 0.809 | 1.000 |
| rs9619311 | 33196693 | C | T | 5/75/256 | 5/67/269 | 0.785 | 0.452 | 0.409 | 1.000 | 1.000 |
| rs135025 | 33202478 | T | C | 86/160/91 | 85/165/89 | 0.664 | 0.957 | 0.862 | 0.930 | 1.000 |
| rs242089 | 33213319 | T | C | 79/160/99 | 76/168/97 | 0.829 | 1.000 | 0.866 | 0.784 | 1.000 |
| rs80272 § | 33224779 | - | T | **0/0/1** | **0/0/1** | **-** | - | - | - | - |
| rs8140818 | 33228722 | C | T | 2/32/308 | 1/41/299 | 1.000 | 0.420 | 0.333 | 1.000 | 1.000 |
| rs715572 | 33234931 | A | G | 34/158/146 | 32/161/147 | 0.222 | 0.908 | 1.000 | 0.797 | 1.000 |
| rs242072 | 33235517 | C | T | 81/160/99 | 79/171/91 | 1.000 | 0.745 | 0.495 | 0.857 | 1.000 |
| rs135029 | 33240290 | A | G | 8/92/240 | 9/79/254 | 0.371 | 0.405 | 0.304 | 1.000 | 1.000 |
| rs1427385 | 33244314 | T | C | 69/168/96 | 72/172/93 | 0.743 | 0.743 | 0.732 | 0.850 | 1.000 |
| rs9609643 | 33251059 | A | G | 3/52/276 | 4/64/267 | 1.000 | 0.231 | 0.232 | 1.000 | 1.000 |
| rs9862 | 33253280 | T | C | 65/161/110 | 57/167/117 | 0.911 | 0.442 | 0.685 | 0.424 | 1.000 |
| rs11547635 | 33253292 | T | C | 41/144/155 | 46/143/153 | 0.187 | 0.689 | 0.878 | 0.647 | 1.000 |
| rs137485 | 33254283 | A | T | 5/89/246 | 7/66/267 | 0.289 | 0.149 | 0.075 | 0.772 | 1.000 |
|  |  |  |  |  |  |  |  |  |  |  |
| ***HMOX1*** |  |  |  |  |  |  |  |  |  |  |
| rs2071749 | 35783413 | A | G | 38/131/169 | 48/148/145 | 0.345 | **0.044** | 0.055 | 0.300 | 1.000 |
| rs2285112 | 35789263 | G | A | 91/163/86 | 75/185/80 | 0.129 | 0.626 | 0.655 | 0.180 | 1.000 |
| rs743811 | 35792974 | T | C | 34/123/179 | 31/140/168 | 0.796 | 0.590 | 0.356 | 0.697 | 1.000 |
|  |  |  |  |  |  |  |  |  |  |  |
| ***PVALB*** |  |  |  |  |  |  |  |  |  |  |
| rs9610583 | 37193986 | C | A | 33/147/161 | 26/139/177 | 1.000 | 0.192 | 0.251 | 0.344 | 1.000 |
| rs4616572 | 37194716 | A | C | 5/336/0 | 3/91/243 | 0.118 | **6.20e-48** | **4.86e-106** | 0.725 | **1.00e-06** |
| rs2413421 | 37194767 | A | C | 20/143/176 | 25/124/189 | 0.477 | 0.622 | 0.317 | 0.445 | 1.000 |
| rs5750309 | 37199022 | C | T | 58/165/115 | 44/175/121 | 0.137 | 0.293 | 0.687 | 0.133 | 1.000 |
| rs2022068 | 37199806 | G | A | 37/146/156 | 14/158/168 | 0.002 | **0.044** | 0.398 | **0.001** | 0.180 |
| rs12171125 | 37201450 | G | A | 8/85/247 | 3/78/259 | 0.448 | 0.206 | 0.334 | 0.223 | 1.000 |
| rs2284021 | 37201817 | T | C | 9/104/226 | 7/87/245 | 1.000 | 0.143 | 0.133 | 0.801 | 1.000 |
| rs4820254 | 37206341 | G | T | 14/120/208 | 5/90/247 | 0.392 | **0.001** | **0.002** | 0.060 | 0.219 |
| rs5750310 | 37208660 | A | C | 31/142/164 | 24/130/184 | 0.889 | 0.116 | 0.144 | 0.329 | 1.000 |
| rs2067069 | 37210469 | G | A | 75/173/94 | 65/176/101 | 0.512 | 0.385 | 0.611 | 0.394 | 1.000 |
| rs2269511 | 37210656 | T | C | 18/144/175 | 22/130/190 | 1.000 | 0.621 | 0.356 | 0.626 | 1.000 |
| rs762920 | 37212536 | A | C | 1/46/292 | 4/51/286 | 0.298 | 0.314 | 0.453 | 0.373 | 1.000 |
| rs2001064 | 37215758 | G | C | 5/86/248 | 6/82/251 | 1.000 | 0.938 | 0.862 | 1.000 | 1.000 |
|  |  |  |  |  |  |  |  |  |  |  |
| ***IL2RB*** |  |  |  |  |  |  |  |  |  |  |
| rs5995385 | 37519864 | C | T | 57/176/107 | 45/172/123 | 0.252 | 0.136 | 0.224 | 0.237 | 1.000 |
| rs228937 | 37520971 | G | T | 44/141/152 | 45/166/128 | 0.489 | 0.156 | 0.061 | 1.000 | 1.000 |
| rs3218339 | 37521211 | A | G | 11/73/254 | 2/80/259 | 0.136 | 0.378 | 0.858 | **0.012** | 0.934 |
| rs228942 § | 37524619 | A | C | 159/1/181 | 146/2/191 | **1.43e-96** | - | - | - | - |
| rs228945 | 37525880 | G | A | 46/144/144 | 34/159/143 | 0.330 | 0.566 | 0.938 | 0.154 | 1.000 |
| rs2072862 | 37528606 | G | A | 54/176/109 | 63/157/121 | 0.372 | 0.912 | 0.373 | 0.417 | 1.000 |
| rs3218312 | 37530332 | C | T | 37/153/149 | 45/148/148 | 0.406 | 0.607 | 0.938 | 0.410 | 1.000 |
| rs228953 | 37531436 | T | C | 53/176/109 | 46/178/115 | 0.090 | 0.507 | 0.683 | 0.449 | 1.000 |
| rs760720 | 37532090 | T | G | 15/138/182 | 17/129/191 | 0.462 | 0.752 | 0.587 | 0.857 | 1.000 |
| rs3218294 | 37533286 | C | G | 11/88/242 | 9/108/224 | 0.471 | 0.283 | 0.162 | 0.821 | 1.000 |
| rs3218292 | 37533530 | G | C | 15/133/192 | 21/123/192 | 0.883 | 0.849 | 0.877 | 0.308 | 1.000 |
| rs228963 | 37535948 | T | C | 43/166/129 | 47/159/135 | 1.000 | 0.955 | 0.753 | 0.735 | 1.000 |
| rs228968 | 37538328 | G | A | 24/135/177 | 24/140/175 | 0.684 | 0.855 | 0.817 | 1.000 | 1.000 |
| rs1003694 | 37539128 | A | G | 47/151/140 | 44/144/152 | 0.280 | 0.426 | 0.395 | 0.736 | 1.000 |
| rs1003693 | 37539188 | T | C | 38/142/157 | 37/137/166 | 0.310 | 0.640 | 0.590 | 0.903 | 1.000 |
| rs2235330 | 37539713 | C | T | 60/169/109 | 71/152/118 | 0.098 | 0.913 | 0.569 | 0.332 | 1.000 |
| rs228973 | 37541812 | C | T | 71/181/88 | 78/160/100 | 0.382 | 0.786 | 0.304 | 0.517 | 1.000 |
| rs228975 | 37542201 | C | T | 31/148/156 | 35/138/166 | 0.444 | 0.814 | 0.538 | 0.698 | 1.000 |
| rs3218258 | 37544245 | T | C | 4/58/277 | 1/78/262 | 0.064 | 0.255 | 0.130 | 0.216 | 1.000 |
| rs2284035 | 37544602 | G | A | 45/157/136 | 53/175/112 | 0.314 | 0.075 | 0.056 | 0.445 | 1.000 |
| rs228979 § | 37544931 | C | A | 0/318/22 | 0/314/26 | **1.14e-69** | - | - | - | - |
|  |  |  |  |  |  |  |  |  |  |  |
| ***SOX10*** |  |  |  |  |  |  |  |  |  |  |
| rs139883 | 38369027 | C | T | 27/137/174 | 20/132/189 | 0.774 | 0.220 | 0.318 | 0.293 | 1.000 |
|  |  |  |  |  |  |  |  |  |  |  |
| ***PDGFB*** |  |  |  |  |  |  |  |  |  |  |
| rs6001508 | 39617702 | A | C | 0/56/286 | 2/52/287 | 1.000 | 1.000 | 0.917 | 0.249 | 1.000 |
| rs9607623 | 39617876 | A | G | 0/51/289 | 2/53/285 | 1.000 | 0.616 | 0.751 | 0.499 | 1.000 |
| rs4416326 | 39623725 | C | T | 23/117/184 | 22/142/169 | 0.341 | 0.261 | 0.137 | 0.878 | 1.000 |
| rs9611119 | 39624248 | T | G | 6/70/263 | 4/81/256 | 0.482 | 0.624 | 0.471 | 0.545 | 1.000 |
| rs4821877 | 39629096 | T | C | 21/132/186 | 33/123/183 | 0.079 | 0.391 | 0.877 | 0.118 | 1.000 |
| rs9622979 | 39629609 | A | G | 3/47/291 | 5/61/274 | 0.390 | 0.091 | 0.104 | 0.505 | 1.000 |
| rs5757573 | 39633622 | C | T | 0/47/293 | 2/46/293 | 0.698 | 0.833 | 1.000 | 0.499 | 1.000 |
|  |  |  |  |  |  |  |  |  |  |  |
| ***ADRBK2*** |  |  |  |  |  |  |  |  |  |  |
| rs133369 § | 42463814 | C | T | **7/50/176** | **1/0/4** | 0.111 | - | - | - | - |
| rs133373 | 42465788 | A | G | 25/132/181 | 23/135/183 | 0.890 | 0.902 | 1.000 | 0.767 | 1.000 |

* The major allele in the control group is designated as allele 1, and minor allele as allele 2.

† SNPs are listed according to their physical positions on chromosome 22 (NCBI build 37.1).

‡ Empirical p values (p_emp_) are estimated based on 10,000,000 permutations. In each round of permutation (swapping of the case-control status), the best *original* result of every SNP is compared against the best result of the three tests (allelic, dominant and recessive) of that SNP, and also against the best results from all SNPs.

§ This marker is discarded because of the p value < 0.001 for HWE testing (controls) or low genotype call rate.
