# Supplementary material for: Association of High Myopia with Crystallin Beta A4 (CRYBA4) Gene Polymorphisms in the Linkage-Identified MYP6 Locus
Source: PLoS One. 2012 Jun 29;7(6):e40238. doi: 10.1371/journal.pone.0040238 (PMC3389832; doi:10.1371/journal.pone.0040238)
Supplement: Appendix S1 — Candidate gene selection from the MYP6 locus and subsequent validation by Endeavour. (DOC) [file pone.0040238.s002.doc]

**Appendix S1**

**Candidate gene selection from the *MYP6* locus and subsequent validation by Endeavour**

Genes were identified from NCBI database and *manually* prioritized into 5 categories – highly relevant (with substantial evidence for ocular disease or function), possibly related (with relatively less evidence for ocular disease or function), unlikely (without evidence for ocular disease or function), homologue genes (genes encoding for homologous proteins), and pseudo, putative or hypothetical genes (genes that are non-functional or not protein-coding). Extensive literature search was done. Genes were given higher credits and corresponding prioritization if there were evidences for relevant biological functions, involvement as structural constituents or in modulation and maintenance of eyes and relationship to ocular diseases.

At the same time, using 14 genes (*LUM*, *HGF*, *MET*, *MYOC*, *PAX6*, *COL1A1*, *COL2A1*, *TGFB1*, *TGIF*, *UMODL1*, *MMP1*, *MMP3*, *MMP9* & *NYX*) [1-22] reported to be associated with myopia as a training set for *in silico* prioritization tool Endeavour [23,24], all genes identified from the *MYP6* locus were prioritized *computationally*. Endeavour integrates information from multiple heterogeneous data sources such as published literature (abstracts from EntrezGene), functional annotation (Gene Ontology), microarray gene expression (Atlas gene expression), EST expression (EST data from Ensembl), protein domain (InterPro), protein-protein interactions (BIND), gene pathway (KEGG), cis-regulatory modules (TOUCAN), transcriptional motifs (TRANSFAC) and sequence similarity (BLAST). Endeavour generates an overall rank based on the fusion of data sources by order statistics.

**Supplementary references**

1. Chen ZT, Wang IJ, Shih YF, Lin LL. (2009) The association of haplotype at the lumican gene with high myopia susceptibility in Taiwanese patients. Ophthalmology 116: 1920-1927.

2. Lin HJ, Kung YJ, Lin YJ, Shen JJ, Chen BH, et al. (2009) Association of the Lumican gene functional 3' UTR polymorphism with high myopia. Invest Ophthalmol Vis Sci 51: 96-102.

3. Wang IJ, Chiang TH, Shih YF, Hsiao CK, Lu SC, et al. (2006) The association of single nucleotide polymorphisms in the 5'-regulatory region of the lumican gene with susceptibility to high myopia in Taiwan. Mol Vis 12: 852-857.

4. Han W, Yap MK, Wang J, Yip SP. (2006) Family-based association analysis of hepatocyte growth factor (HGF) gene polymorphisms in high myopia. Invest Ophthalmol Vis Sci 47: 2291-2299.

5. Yanovitch T, Li YJ, Metlapally R, Abbott D, Viet KN, et al. (2009) Hepatocyte growth factor and myopia: genetic association analyses in a Caucasian population. Mol Vis 15: 1028-1035.

6. Tang WC, Yip SP, Lo KK, Ng PW, Choi PS, et al. (2007) Linkage and association of myocilin (MYOC) polymorphisms with high myopia in a Chinese population. Mol Vis 13: 534-544.

7. Vatavuk Z, Skunca Herman J, Bencic G, Andrijevic DB, Lacmanovic LV, et al. (2009) Common variant in myocilin gene is associated with high myopia in isolated population of Korcula Island, Croatia. Croat Med J 50: 17-22.

8. Wu H, X.H. Y, Yap E. (1999) Allelic association between trabecular meshwork-induced glucocorticoid response (TIGR) gene and severe sporadic myopia. Invest Ophthalmol Vis Sci 40: S600.

9. Wu H, Yong R, Tan S, Yap E. (2000) Further evidence of association between trabecular meshwork-induced glucocorticoid response (TIGR/Myocilin) gene and severe myopia. Invest Ophthalmol Vis Sci 41: S33.

10. Han W, Leung KH, Fung WY, Mak JY, Li YM, et al. (2009) Association of PAX6 polymorphisms with high myopia in Han Chinese nuclear families. Invest Ophthalmol Vis Sci 50: 47-56.

11. Ng TK, Lam CY, Lam DS, Chiang SW, Tam PO, et al. (2009) AC and AG dinucleotide repeats in the PAX6 P1 promoter are associated with high myopia. Mol Vis 15: 2239-2248.

12. Tsai YY, Chiang CC, Lin HJ, Lin JM, Wan L, et al. (2008) A PAX6 gene polymorphism is associated with genetic predisposition to extreme myopia. Eye 22: 576-581.

13. Metlapally R, Li YJ, Tran-Viet KN, Abbott D, Czaja GR, et al. (2009) COL1A1 and COL2A1 genes and myopia susceptibility: evidence of association and suggestive linkage to the COL2A1 locus. Invest Ophthalmol Vis Sci 50: 4080-4086.

14. Mutti DO, Cooper ME, O'Brien S, Jones LA, Marazita ML, et al. (2007) Candidate gene and locus analysis of myopia. Mol Vis 13: 1012-1019.

15. Lin HJ, Wan L, Tsai Y, Tsai YY, Fan SS, et al. (2006) The TGFbeta1 gene codon 10 polymorphism contributes to the genetic predisposition to high myopia. Mol Vis 12: 698-703.

16. Zha Y, Leung KH, Lo KK, Fung WY, Ng PW, et al. (2009) TGFB1 as a susceptibility gene for high myopia: a replication study with new findings. Arch Ophthalmol 127: 541-548.

17. Lam DS, Lee WS, Leung YF, Tam PO, Fan DS, et al. (2003) TGFbeta-induced factor: a candidate gene for high myopia. Invest Ophthalmol Vis Sci 44: 1012-1015.

18. Inamori Y, Ota M, Inoko H, Okada E, Nishizaki R, et al. (2007) The COL1A1 gene and high myopia susceptibility in Japanese. Hum Genet 122: 151-157.

19. Nishizaki R, Ota M, Inoko H, Mequro A, Shiota T, et al. (2009) New susceptibility locus for high myopia is linked to the uromodulin-like 1 (UMODL1) gene region on chromosome 21q22.3. Eye 23: 222-229.

20. Hall NF, Gale CR, Ye S, Martyn CN. (2009) Myopia and polymorphisms in genes for matrix metalloproteinases. Invest Ophthalmol Vis Sci 50: 2632-2636.

21. Khor CC, Grignani R, Ng DP, Toh KY, Chia KS, et al. (2009) cMET and refractive error progression in children. Ophthalmology 116: 1469-1474.

22. Zhang Q, Xiao X, Li S, Jia X, Yang Z, et al. (2007) Mutations in NYX of individuals with high myopia, but without night blindness. Mol Vis 13: 330-336.

23. Aerts S, Lambrechts D, Maity S, Van Loo P, Coessens B, et al. (2006) Gene prioritization through genomic data fusion. Nat Biotechnol 24: 537-544.

24. Tranchevent LC, Barriot R, Yu S, Van Vooren S, Van Loo P, et al. (2008) ENDEAVOUR update: a web resource for gene prioritization in multiple species. Nucleic Acids Res 36: W377-W384.
